# A new genus and two new species of South American Myodochini (Hemiptera, Heteroptera, Rhyparochromidae)

**DOI:** 10.3897/zookeys.796.21431

**Published:** 2018-11-15

**Authors:** Pablo M. Dellapé, María Cecilia elo, Sara I. Montemayor

**Affiliations:** 1 Universidad Nacional de La Plata, CONICET, División Entomología, Museo de La Plata, Paseo del Bosque s/n B1900FWA, La Plata, Buenos Aires, Argentina Universidad Nacional de La Plata La Plata Argentina

**Keywords:** *
Henryaria
*, *
Henryaria thomasi
*, *
Henryaria zongo
*, Lygaeoidea, Rhyparochrominae

## Abstract

The new Neotropical genus *Henryaria* (Heteroptera, Rhyparochromidae, Myodochini) is established to accommodate two new species from Bolivia and Peru. Photographs of the types and their male genitalia are provided. Similarities with other genera of the tribe are discussed, as well as the characters to distinguish the two new species.

## Introduction

The Rhyparochromidae are the most diverse group of the Lygaeoidea with more than 2,000 species (Henry et al. 2015). It is divided into two subfamilies Plinthisinae and Rhyparochrominae, the latter of which includes 14 tribes (Henry 1997).

The Myodochini comprise 77 extant genera and 368 species worldwide; 38 genera and 121 species are known from the Neotropics (Dellapé and Henry 2017). Many species live on the ground, where they feed on fallen seeds. The species that ascend plants to feed are the most commonly collected myodochines. Other species live in forest canopies (Dellapé and Henry 2010, Henry et al. 2015). As most rhyparochromids do, the members of this tribe feed on mature seeds. Many species are myrmecomorphic, and in some cases, although they are not morphologically similar to ants, both adults and nymphs mimic them in their movements (Slater and Baranowski 1990).

Phylogenetic relationships within the tribe are not clear. The only attempt to establish a cladistic framework is Harrington’s (1980) analysis, where four main groups, representing 56 genera, were established based mainly on characters of the male genitalia. Since then, new genitalic studies and the discovery of new taxa have led to a need for a taxonomic update and reevaluation of relationships.

The actual diversity of the group is much higher than the current numbers indicate (Henry et al. 2015). For example, in the recent revision of the Neotropical genus *Heraeus*, 30 new species and two new genera were described (Dellapé et al. 2016).

In the present contribution a new genus is described to include two new species from Bolivia and Peru.

## Materials and methods

Color images were captured using a digital camera (Micrometrics 391CU, 3.2 m) mounted on a Nikon SMZ1000 stereomicroscope. Multiple focal planes were merged using Micrometrics SE Premium 4 software.

The genital structures were dissected under a stereomicroscope, cleared in a 10% KOH aqueous solution, washed in distilled water, and preserved in a vial with glycerin. All measurements are in millimeters. The acronyms used are **USNM** for the National Museum of Natural History, Washington, D.C., USA, and **MLP** for the Museo de la Plata, La Plata, Argentina.

## Taxonomy

### Tribe Myodochini

#### Henryaria gen. n.

Taxon classificationAnimaliaHemipteraRhyparochromidae

http://zoobank.org/51EF789F-24B6-40C3-8167-32A9FB387D56

Figures 1–5 , 6–10 , 11–14 , 15–19

##### Type species.

*Henryariathomasi* sp. n.

##### Diagnosis.

Head strongly convex behind eyes, forming short neck; eyes relatively small, not surpassing dorsal margin of head; jugal ridge developed; vertex rounded; buccular juncture V-shaped. Evaporative area extensive. Mesepimeron emergent. Profemur incrassate, with two rows of spines; aedeagus without spines, seminal duct on vesica and gonoporal process distinctly wide; gonoporal process broadened towards apex.

##### Description.

**Figures 1–5.**
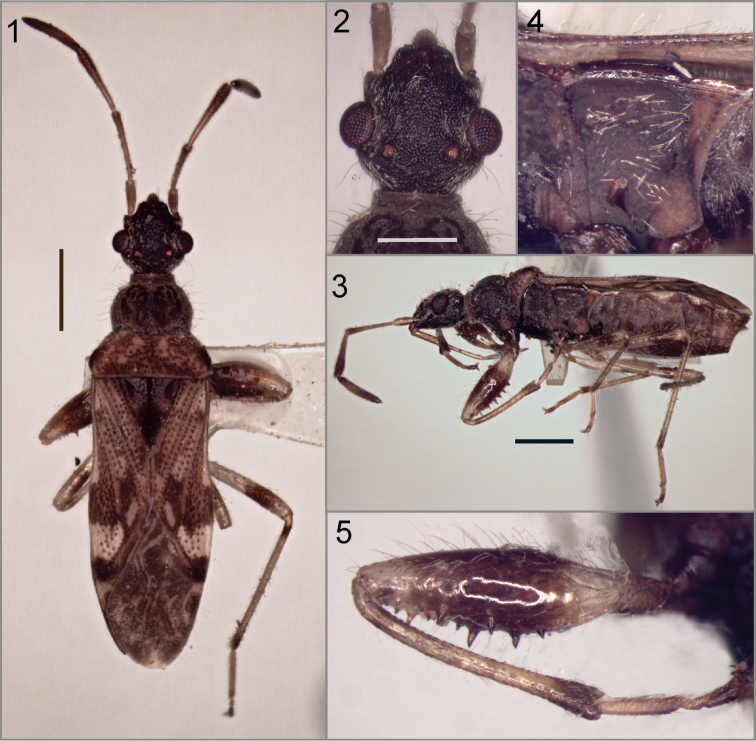
*Henryariathomasi* sp. n. **1** dorsal view **2** head **3** lateral view **4** metapleuron **5** anterior leg. Scale bars: 1 mm (**1, 3**); 0.5 mm (**2**).

Relatively small (ca. 6 mm long), pilose. *Head* (Figs 1–3, 11–14) shiny, with many grouped punctures forming a coriaceous texture; head strongly convex behind eyes, forming a short neck; eyes relatively small, not surpassing dorsal margin of head in lateral view; ocelli closer to eyes than to posterior margin of head; jugal ridge developed; vertex rounded; buccular juncture V-shaped at level of antenniferous tubercles. Scape relatively short but surpassing apex of head.

*Thorax*: Pronotum, scutellum and hemelytra pruinose. Pronotum punctate, with punctures slightly larger on posterior pronotal lobe; lateral margins of both pronotal lobes rounded; with ring-like collar well differentiated. Clavus with 3 rows of punctures, and partial fourth between inner and median rows on distal three-quarters. Evaporative area extensive. Mesepimeron emergent (Fig. 4). Procoxa with spine; protrochanter unarmed; profemur (Figs 5, 13) incrassate, with 2 rows of spines; protibia slightly curved, with numerous minute tubercles over entire ventral surface; male mesofemur unarmed.

*Aedeagus* (Figs 10, 19) lacking spines, sperm reservoir well developed, vesica with two membranous lobes partially sclerotized; seminal duct on vesica and gonoporal process distinctly wide; gonoporal process broadened towards apex.

##### Etymology.

This new genus is named after our dear friend Thomas J. Henry (Systematic Entomology Laboratory [SEL], ARS, USDA, c/o National Museum of Natural History, Washington, DC), in honor of his many fundamental contributions to the knowledge of Heteroptera. Besides his brilliant career, Dr. Henry has been a role model to us, always sharing his knowledge and passion for true bugs.

#### Henryaria thomasi sp. n.

Taxon classificationAnimaliaHemipteraRhyparochromidae

http://zoobank.org/EB39D9B4-8A09-4D47-8E6A-CFCC45D33BE1

Figures 1–5 , 6–10

##### Material examined.

Holotype male, Peru, Satipo, IX-10-1941, P. Paprzychi, J. C. Lutz collection (USNM). Paratype male, same data (USNM). Paratype female, same data, IX-24-1941 (MLP).

##### Description.

***Male holotype***. Total length 5.89. Head length 1.04, width 1.03. Postocular length 0.29. *Head* (Figs 1–3) reddish brown, clypeus paler; with long erect and semierect setae directed dorsally; ocelli at level of imaginary line across posterior margin of eyes. Interocular space 0.55, interocellar space 0.30. Antennal lengths: scape 0.48, pedicel 0.97, basiflagellomere 0.86, distiflagellomere 1.00. Antennae light brown, with distal region of pedicel and basiflagellomere darker; distiflagellomere with a diffuse paler band basally occupying half of segment; basiflagellomere slightly clavate; with numerous short recumbent setae and sparse erect setae. Labial segment lengths: I 0.62, II 0.66, III 0.38, IV 0.40. Labium light brown with sparse erect setae, surpassing procoxae (Fig. 3).

*Thorax*: Collar length 0.08, anterior lobe length 0.64, posterior lobe length 0.55; anterior lobe width 1.00, posterior lobe width 1.60. Anterior pronotal lobe dark brown, posterior lobe brown with irregular paler areas, humeral angles darker; with long erect setae on both lobes. Pleurae dark brown, paler on pro- and metaepimeron (Fig. 4); with semierect and erect setae. Scutellum dark brown, with long erect setae. Hemelytron (Fig. 1) with numerous long erect setae; corial margin smooth; clavus light brown; corium irregularly pigmented with darker transverse band across middle and apical region; membrane dark with pale macula at apex, veins partially paler. Coxae, protrochanter, profemur (Fig. 5) except base and apex, distal band on meso- and metafemur, a basal short band on tibiae (diffuse on protibia), tibiae and tarsi distally and pretarsi brown, rest of legs light brown. Femora and tibiae with numerous erect and semierect setae.

**Figures 6–10.**
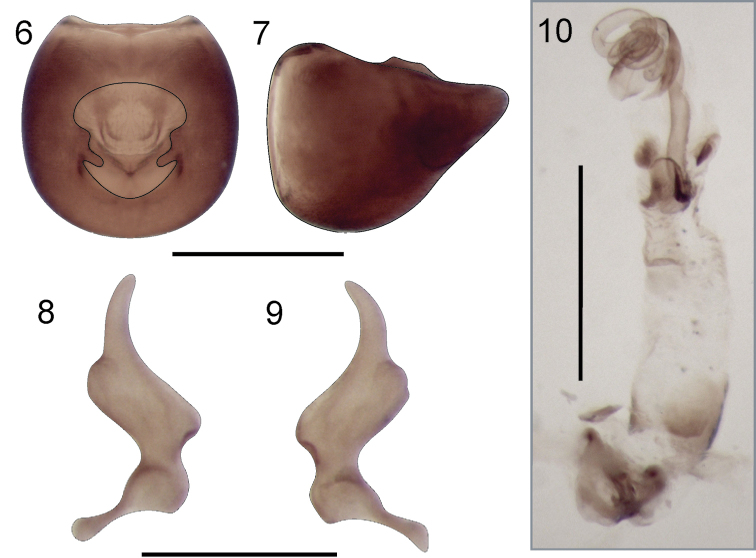
*Henryariathomasi* sp. n. **6–7** Pygophore **6** dorsal view **7** lateral view. **8–9** Parameres **8** inner view **9** outer view. **10** Aedeagus. Scale bars: 0.5 mm (**6, 7, 10**); 0.25 mm (**8, 9**).

*Abdomen* brown with paler areas on lateral margin of segments V and VI; with numerous recumbent, erect and semierect setae. Male genitalia: Pygophore (Figs 6–7) broadly rounded, anterior margin of dorsal aperture rounded, inner projections produced posteriorly. Parameres: blade relatively short, outer projection broadly rounded (Figs 8–9). Aedeagus (Fig. 10) without spines, vesica with two lobes partially sclerotized; seminal duct on vesica wide; gonoporal process broadened towards apex.

Paratypes: As holotype description, except male paratype (Figs 1–2) with distiflagellomere unicolorous; and female paratype with posterior margin of posterior pronotal lobe darker.

Measurements of male and female paratype, respectively: Total length 5.70/5.89. Head length 1.03/1.08, width 0.98/1.03. Postocular length 0.24/0.29. Interocular space 0.55/0.59, interocellar space 0.29/ 0.30. Labial segment lengths: I 0.59/0.65, II 0.66/0.67, III -/0.41, IV -/0.36. Antennal lengths: Scape 0.48/0.48, pedicel 0.92/, basiflagellomere 0.86/-, distiflagellomere 1.01/-. Collar length 0.08/0.08, anterior lobe length 0.65/0.60, posterior lobe length 0.55/0.60; anterior lobe width 1.00/1.04, posterior lobe width 1.52/1.60.

##### Etymology.

We are pleased to dedicate this new species to Thomas Henry in recognition of his invaluable contributions to the knowledge of Heteroptera.

#### Henryaria zongo sp. n.

Taxon classificationAnimaliaHemipteraRhyparochromidae

http://zoobank.org/A8259FB9-5161-46A6-9109-8AFF55ADE624

Figures 11–14 , 15–19

##### Material examined.

Holotype male, Bol.[ivia], La Paz, Rio Zongo, 1400 m, 24/30-X-84, L. E. Pena coll. (USNM).

##### Description.

**Figures 11–14.**
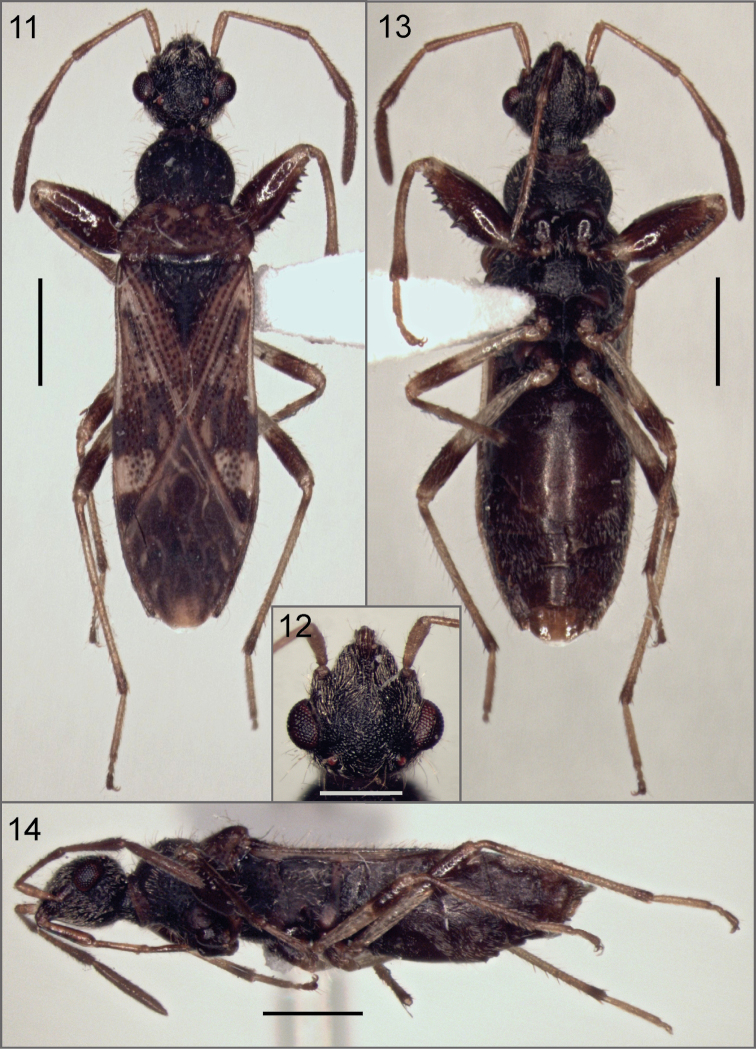
*Henryariazongo* sp. n. **11** dorsal view **12** head **13** ventral view **14** lateral view. Scale bars: 1 mm (**11, 13, 14**); 0.5 mm (**12**).

***Male Holotype***. Total length 5.60. Head length 0.94, width 1.04; postocular length 0.24. *Head* (Figs 11, 12, 14) strongly globose; fuscous except clypeous pale brown; with numerous long erect and semierect forward-directed setae, postocular region with same setae but directed backward; eyes oval; ocelli placed just behind imaginary line across posterior margin of eyes. Interocular space 0.62, interocellar space 0.38. Antennal lengths: Scape 0.42, pedicel 0.85, basiflagellomere 0.72, distiflagellomere 0.92. Antennae light brown, distal region of pedicel and basiflagellomere and entire distiflagellomere darker; basiflagellomere slightly clavate; with numerous short recumbent setae and sparse erect setae. Labial segment lengths: I 0.6, II 0.58, III 0.40, IV 0.36. Labium light brown with sparse erect setae, reaching procoxae (Fig. 14).

*Thorax*: Collar length 0.06, anterior lobe length 0.65, posterior lobe length 0.49; anterior lobe width 1.01, posterior lobe width 1.42. Anterior pronotal lobe fuscous, posterior lobe brown with four irregular light brown maculae (Fig. 11); with long erect setae on both lobes. Pleura dark brown, paler on pro- and metaepimeron; with semierect and erect setae. Scutellum fuscous, with long erect setae. Hemelytron (Fig. 11) with numerous long erect setae; corial margin smooth with numerous setae on anterior half; clavus brown darker basally next to scutellum; corium irregularly pigmented with darker transverse band across middle and apical region; membrane dark with veins partially paler. Coxae, protrochanter, profemur (Fig. 13) except extreme base and apex, distal band on meso- and metafemur, basal band on meso- and metatibia, tibiae distally and pretarsi brown, rest of legs light brown. Femora and tibiae with numerous erect and semierect setae.

**Figures 15–19.**
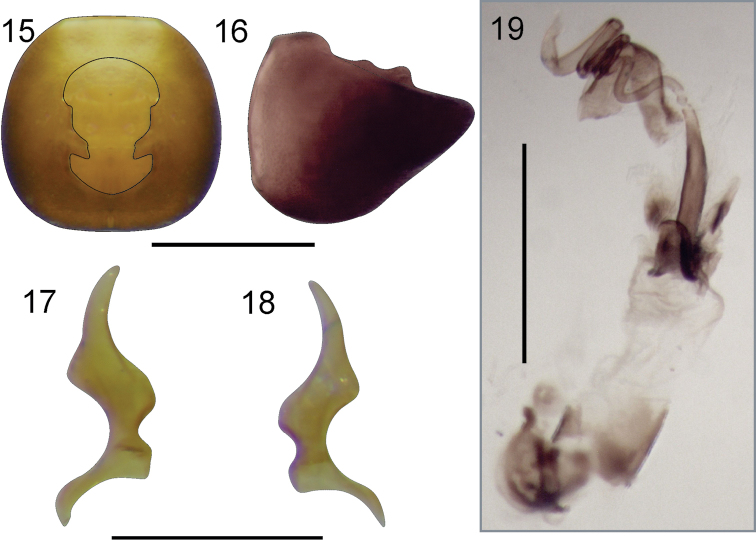
*Henryariazongo* sp. n. **15–16** Pygophore **15** dorsal view **16** lateral view. **17–18** Parameres **17** inner view **18** outer view. **19** Aedeagus. Scale bars: 0.5 mm (**15, 16, 19**); 0.25 mm (**17, 18**).

*Abdomen* brown, with numerous recumbent, erect and semierect setae. Male genitalia: Pygophore (Figs 15–16) broadly rounded, anterior margin of dorsal aperture rounded, inner projections produced posteriorly. Paramere: blade relatively long (Figs 17–18) Aedeagus (Fig. 19) without spines, vesica with two lobes partially sclerotized; seminal duct on vesica wide; gonaporal process broadened toward apex.

##### Etymology.

The specific epithet refers to the river where the specimen was collected.

## Discussion

*Henryaria* gen. n. runs to couplet 35 in Harrington’s (1980) key to the Myodochini of the world, and to couplet 26 in the key by Henry et al. (2015) to the Neotropical genera of Myodochini. In both keys the genera *Neopamera* Harrington, 1980 and *Orthaea* Dallas, 1852 are recognized. *Neopamera* was erected by Harrington (1980) to include several New World species that lack synapomorphies for the genus. The type species, *N.bilobata* (Say, 1831), has the following character states useful for generic diagnosis: postocular region of head wide not forming neck; male protrochanters with small spine and procoxa with two large spines, and females with profemora less incrassate, trochanters without spines and single spine on procoxa; seminal duct of vesica and gonoporal processes slender. In contrast, the genus *Orthaea* includes large species (Dellapé and Montemayor 2008) ranging from 8.4 to 10.5 mm long. In addition to the slightly elongate head placed at a lower plane than the posterior lobe, as mentioned by Harrington (1980), the species of *Orthaea* are defined by the long scape, at least as long as the head, and the elongated and slightly stout male profemur (Dellapé and Montemayor 2011). Since the key to the Neotropical genera of Myodochini appeared (Henry et al. 2015), two new genera have been described (Dellapé et al. 2016): *Baranowskiobius* Dellapé, Melo and Henry and *Paraheraeus* Dellapé, Melo and Henry. The species included in both genera are larger and more slender (ca. 7 to > 10 mm long), with a postocular region longer than the interocellar length, and less convex, not abruptly constricted, forming a distinct but short neck as in *Henryaria* species.

The two new species included in *Henryaria* are similar in general aspect and color patterns, but they can be distinguished by the shape of the head. *Henryariazongo* sp. n. presents a shorter and globose head, with a strongly convex dorsal region; the labium is shorter with segment four in resting position between procoxae; and the profemur is more incrassate and almost entirely brown except the extreme base and apex. In contrast, *H.thomasi* sp. n. has a more elongate head; the labium is longer, extending beyond the procoxae; the profemur is less incrassate and the basal and apical pale areas of the profemur are more extended. The male genitalia are similar in both species, but the parameres show differences in the length of the blade (shorter in *H.thomasi*) and in the shape of the inner projection of the dorsal aperture of the pygophore.

## Supplementary Material

XML Treatment for Henryaria

XML Treatment for Henryaria thomasi

XML Treatment for Henryaria zongo
